# Relationship Between Greyscale Ultrasound Grading of Hepatic Steatosis and Attenuation Imaging

**DOI:** 10.7759/cureus.23435

**Published:** 2022-03-24

**Authors:** Abdur Rehman, Jaideep Darira, Kamran Hamid, Muhammad Saad Ahmed, Muhammad Kashif Shazlee, Ashraf Amirali

**Affiliations:** 1 Diagnostic Radiology, Dr. Ziauddin Hospital, Karachi, PAK; 2 Imaging Services, Indus Hospital and Health Network, Karachi, PAK; 3 Diagnostic Radiology, National Institute of Child Health, Karachi, PAK

**Keywords:** greyscale ultrasound, non-alcoholic fatty liver disease, attenuation imaging, greyscale, hepatic steatosis

## Abstract

Background

Non-alcoholic fatty liver disease (NAFLD) has been rising worldwide due to the rising public health threat of metabolic syndrome. Because non-alcoholic steatohepatitis can proceed to liver fibrosis and cirrhosis, early identification and monitoring are critical for management. For the examination of NAFLD, greyscale ultrasound has been frequently employed. A relatively new technique, attenuation imaging (ATI), can quantitatively evaluate and compute the attenuation coefficient (AC). Our goal was to evaluate the performance and cutoff values of attenuation imaging to identify hepatic steatosis. As a reference standard, greyscale ultrasound was employed.

Method

A total of 207 patients were assessed from June to November 2021 after getting informed consent. The association between ATI values and greyscale grading to diagnose hepatic steatosis was investigated, and the Statistical Package for the Social Sciences (SPSS) version 21 (IBM Corp., Armonk, NY, USA) was used to analyze the data. In the analysis, the Spearman correlation and area under the receiver operating characteristic curve (AUROC) tests were performed. Receiver operating characteristic curve analysis was also used to assess ATI’s diagnostic capability and cutoff values.

Result

The correlation between ATI values and hepatic steatosis grades on greyscale was statistically significant (p < 0.05). Greyscale grading and ATI levels have a correlation coefficient (r) of 0.85, indicating a strong association. Steatosis grades 1, 2, and 3 had threshold ATI values of 0.65, 0.73, and 0.96 dB/cm/MHz, respectively. According to greyscale, the diagnostic ability of ATI for steatosis grades 1, 2, and 3 were 0.948 (95% CI: 0.917-0.979), 0.978 (95% CI: 0.961-0.995), and 1.000 (95% CI: 1.000-1.000), respectively.

Conclusions

Attenuation imaging is a reliable method for identifying liver steatosis, with great performance and a strong association with the greyscale ultrasound.

## Introduction

Non-alcoholic fatty liver disease (NAFLD) is a commonly found condition that can damage hepatic parenchyma because of associated fibrotic changes and inflammation, resulting in increased parenchymal rigidity of the liver [1].

Globally, the prevalence of NAFLD was found to be 24% [2]. Although the Middle East and South America are significant contributors, Asia follows closely [3]. Considering South Asia only, the prevalence rate ranges from 13% to 34%. Although Pakistan ranks the lowest with a 13% prevalence rate, NAFLD’s increasing severity and prevalence are alarming [4]. According to the Japanese Society of Gastroenterology, early diagnosis of very mild fatty liver (5%-10% on histopathology) is significant for early patient management [5].

Liver biopsy is considered a gold standard [6]. However, due to many shortcomings, such as the invasiveness of the procedure, error in tissue sampling, risk of bleeding, and inter-reader variability, it is no longer a physician’s first choice. It has now been replaced with more non-invasive methods. According to the European Association for the Study of the Liver [7], greyscale ultrasonography should be done before any other modality for diagnosing hepatic steatosis because of its inexpensiveness and wide availability.

A relatively advanced and new technique was brought to us by Canon Medical Systems on the Aplio i-series systems (Canon Medical Systems Corporation, Tochigi, Japan) to diagnose and quantify hepatic steatosis, which is called attenuation imaging (ATI). It is an application that is applied in greyscale ultrasound examination, offering an attenuation coefficient (AC) in tissues, which is presented in a 2D color map of ultrasound [8].

Since attenuation imaging is a new technique, not many studies have been published [9]. Because of variances in the patient population, the cutoff attenuation coefficient, and area under the receiver operating characteristic curve (AUROC) values for recognizing each degree of hepatic steatosis differed from study to study [10]. We evaluated the association between attenuation imaging and the greyscale grading of hepatic steatosis in our population. The performance and cutoff values of attenuation imaging to identify hepatic steatosis using greyscale ultrasound as a reference standard were also assessed.

## Materials and methods

This prospective study was conducted exclusively at Dr. Ziauddin University and Hospital, Karachi, Pakistan. After getting approval from the Ethical Review Committee, data of 207 outpatients and inpatients were collected from June 2021 to November 2021. Non-probability convenient sampling was used. The inclusion criterion for the research was patients between ages 18 and 80 years who provided us with informed consent. Patients with space-occupying lesions in the liver, cirrhotic liver, ascites, and alcohol consumption history were excluded.

In order to evaluate the severity of hepatic steatosis on greyscale ultrasound images, the following gradings were used [1]: grade 0: echogenicity of the liver is average, showing no signs of steatosis; grade 1: mild increase in liver echogenicity, showing normal echogenicity of the portal vein wall and diaphragm; grade 2: moderate increase in liver echogenicity, showing reduced echogenicity of the portal vein wall and diaphragm; and grade 3: severe increase in echogenicity of the liver with near to no visualization of the portal vein wall and diaphragm.

The Canon Aplio i800 machine was utilized. Patients were required to fast six hours before the examination. For the grading of hepatic steatosis, greyscale B-mode imaging was used. Then, attenuation imaging was used by placing a probe through the right intercostal approach. The patient was requested to hold breath, and a sampling box was placed in the liver parenchyma about 2 cm under the liver capsule to avoid reverberation artifacts. Inside the center of the sampling box, about 2 × 4 cm sized region of interest was positioned. Five values of the attenuation coefficient were taken, and the mean of these values was used to grade hepatic steatosis.

Statistical analysis

The Statistical Package for the Social Sciences (SPSS) version 21 (IBM Corp., Armonk, NY, USA) was used to analyze the data. The relationship between greyscale and attenuation imaging was evaluated using Spearman’s rho. To evaluate the diagnostic performance of attenuation imaging and the accuracy of the technique, sensitivity, specificity, and cutoff values, the receiver operating characteristic curve was calculated. p < 0.05 was considered statistically significant.

Funding

The authors did not obtain any financial assistance for this project.

## Results

A total of 207 participants were enrolled for the study from June to November 2021. The majority (61.8%) were male, and 38.2% were females. The participants’ age was from 18 to 77, with a mean age of 43 years.

Based on the gradings of greyscale ultrasound, a large proportion was of grade 0 (48.3%). Grades 1 and 2 were 29.95% and 20.28%, respectively. For grade 3, only 1.44% was found, as shown in Table 1.

**Table 1:** Frequencies of different levels of greyscale

Greyscale levels	N (%)
Normal	100 (48.3)
Mild	62 (29.95)
Moderate	42 (20.28)
Severe	3 (1.44)
Total	207 (100)

Attenuation imaging values based on hepatic steatosis grading on greyscale ultrasound

**Figure 1:**
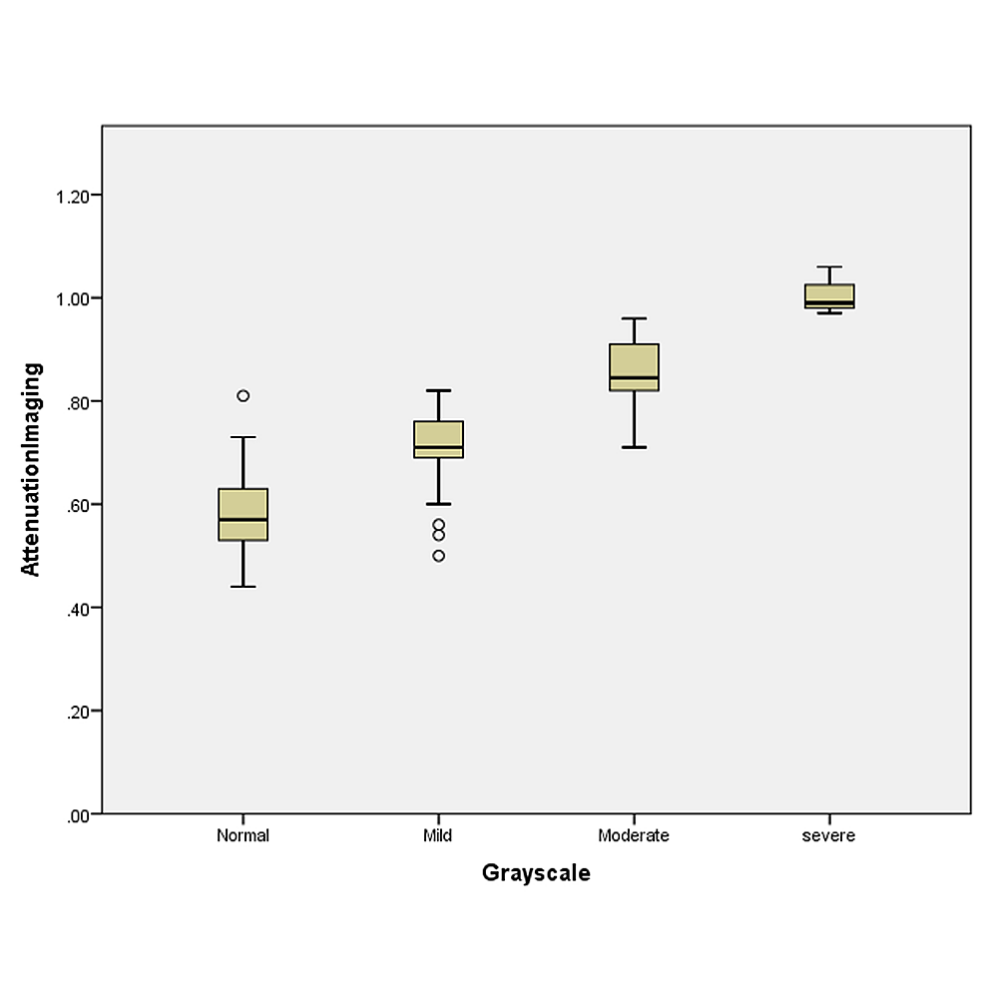
Relationship between attenuation imaging values and steatosis grades based on greyscale ultrasound

Figure 1 demonstrates the relationship between greyscale ultrasound and attenuation imaging values where the ATI values increase with the increase in the grades of hepatic steatosis on greyscale ultrasound.

The mean attenuation values and interquartile ranges are depicted in Table 2.

**Table 2:** Interquartile ranges on attenuation imaging

Sonographic grades	Means ± SE (minimum–maximum)	Interquartile ranges
Grade 0	0.58 ± 0.006 (0.44–0.81)	0.53–0.63
Grade 1	0.71 ± 0.007 (0.50–0.82)	0.69–0.76
Grade 2	0.85 ± 0.010 (0.71–0.96)	0.82–0.91
Grade 3	1.00 ± 0.027 (0.97–1.06)	0.98–1.02

Correlation between greyscale and attenuation imaging

The correlation between attenuation imaging and greyscale is statistically significant, where the value of r was 0.845 (p = 0.000).

Diagnostic performance of attenuation imaging

**Figure 2:**
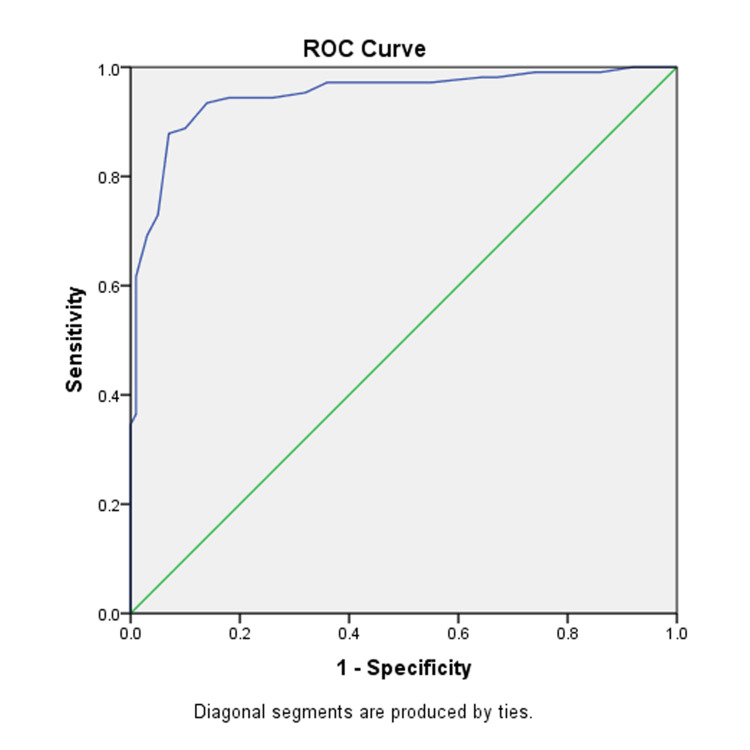
ROC curve for mild fatty liver

Figure 2 is the graphical presentation of the ROC curve of analysis of grade 1 on attenuation imaging, as determined with the help of greyscale ultrasound. The value of AUROC stands at 0.948, a significant diagnostic value. The sensitivity, specificity, and cutoff values are 0.94, 0.82, and 0.65 dB/cm/MHz, respectively.

The ROC curve for the diagnosis of grade 2 with the AUROC value of 0.978 also shows a high diagnostic value. The sensitivity, specificity, and cutoff values are 0.956, 0.85, and 0.73 dB/cm/MHz, respectively.

As the data shows only three cases of patients with grade 3 hepatic steatosis, the AUROC for this is 1.00, with sensitivity, specificity, and cutoff values of 1.00, 1.00, and 0.96 dB/cm/MHz, respectively. This is summarized in Table 3.

**Table 3:** AUROC values of attenuation imaging

Model	Steatosis stage	AUROC (95% CI)	Cutoff	Sensitivity	Specificity
ATI	Stage 1	0.948 (0.917–0.979)	0.65	0.944	0.82
	Stage 2	0.978 (0.961–0.995)	0.73	0.956	0.85
	Stage 3	1.000 (1.000–1.000)	0.96	1.000	1.00

## Discussion

The current study showed a statistically significant relationship between ATI values and greyscale grading of hepatic steatosis. Moreover, the correlation between greyscale Ultrasound and ATI values was >0.8, indicating a strong association. The AUROC of the ATI for hepatic steatosis grade 1 based on greyscale grading was >0.9 in ROC analysis, indicating a high diagnostic value. In addition, the AUROCs of this technique were also >0.9 for both steatosis grades 2 and 3. As a result, ATI is thought to have great non-invasive diagnostic capabilities for quantifying hepatic steatosis.

Our results were similar to those of Yoo et al. who presented that there was a strong link between attenuation imaging and greyscale grades of hepatic steatosis [8]. They also showed that ATI had high test-retest reliability in suspected patients with hepatic steatosis and strong inter-observer agreement in asymptomatic volunteers.

The controlled attenuation parameter (CAP) has played a vital role in quantifying hepatic steatosis for more than a decade. Measurements are taken using M and XL probe, with an area of ​​ interest that is cylindrical. In addition, the probe is placed in a limited area on the skin as recommended by the manufacturer. This technique does not give B-mode liver imaging and restricts the evaluation to a particular area but provides standardized measurements. In contrast to CAP, the attenuation coefficient measurements with attenuation imaging are accompanied by B-mode imaging. It enables the selection of the better region for calculation and to rule out other reasons for liver disease and display the change in echogenicity of liver parenchyma associated with hepatic steatosis; it also has a considerably higher measuring range than CAP [9].

In a study done by Lee et al., the AUROC of ATI’s AC for detecting grade 1 was 0.93 and appeared to be similar to CAP from earlier research [11]. CAP values were less significant in patients with heterogeneous hepatic steatosis and obese patients.

Yoo et al. found that skin thickness and BMI do not influence ATI values as ATI values use only reliable areas on the ATI map [8].

In the research by Bae et al., the AC values obtained from ATI have a strong relationship with the grades of liver steatosis as determined by histopathology (0.660, p < 0.001). The technique was applied to all patients, showing that ATI was highly applicable clinically [12].

Tada et al. also found that the ATI values for each degree of fatty liver were positively correlated with histologically proven grades of fatty liver (p < 0.001). The ATI AUROC for grade 1 liver steatosis showed a moderate diagnostic value (>0.8). For the second and third grades of hepatic steatosis, AUROCs were of a higher diagnostic value (>0.9). These results were significant as the liver steatosis of these patients was diagnosed histologically [13]. Furthermore, these values were assessed in patients with non-alcoholic fatty liver disease and patients with other causes.

Another research showed a statistically significant trend toward a higher attenuation imaging value with higher grades of steatosis, as was obtained from the proton density fat fraction (PDFF) values of MRI [14].

Lee assessed studies based on the quantification of hepatic steatosis using attenuation imaging [10]. Six of them used biopsy, and four have MRI-PDFF values as the reference standard. The cutoff attenuation coefficient values and the AUROC values for identifying each degree of hepatic steatosis varied from study to study, most likely because of the differences in the patient population, liver disease etiology, and hepatic steatosis grade distribution. According to all studies, the attenuation coefficient evaluated by ATI increased with the progression of hepatic steatosis degree. The AUROC score for identifying grade 1 hepatic steatosis using the attenuation coefficient ranged from 0.76 to 0.97, with a cutoff value of 0.59-0.69. With a cutoff attenuation coefficient of 0.67-0.78, the AUROC score for identifying grade 2 hepatic steatosis ranged from 0.86 to 0.99. With an AUROC value of the attenuation coefficient to identify grade 3 hepatic steatosis ranging from 0.79 to 0.97 and a cutoff value of 0.68-0.86, the ATI method worked well in identifying grade 3 steatosis.

The AUROC score for identifying hepatic steatosis using the attenuation coefficient for grade one ranged from 0.76 to 0.97; for grade two, 0.86 to 0.99; and for grade three, 0.79 to 0.97. The cutoff values for grades 1, 2, and 3 ranged from 0.59-0.69, 0.67-0.78, and 0.68-0.86, respectively.

Our study has a few limitations. We used qualitative evaluation of B-mode ultrasound rather than a pathologic evaluation of hepatic steatosis to correlate the ATI results. Moreover, the diagnostic accuracy for attenuation imaging for grade 3 hepatic steatosis was limitedly assessed as we had only three patients.

## Conclusions

In conclusion, our results show that the new technique, attenuation imaging, an objective, non-invasive imaging tool, is a reliable method for detecting hepatic steatosis. It strongly correlates with greyscale ultrasound, with great performance. In the future, attenuation imaging may replace CAP and serve as a quantitative alternative to greyscale ultrasonography. Different cutoff values of attenuation imaging for different grades have been recommended in other studies; therefore, further studies with diverse ethnicities are needed.
